# Genome Sequences of Novel Torque Teno Viruses Identified in Human Brain Tissue

**DOI:** 10.1128/MRA.00924-20

**Published:** 2020-09-10

**Authors:** Simona Kraberger, Diego Mastroeni, Elaine Delvaux, Arvind Varsani

**Affiliations:** a The Biodesign Center for Fundamental and Applied Microbiomics, School of Life Sciences, Arizona State University, Tempe, Arizona, USA; b The Biodesign ASU-Banner Neurodegenerative Disease Research Center, Arizona State University, Tempe, Arizona, USA; c School of Life Sciences, Arizona State University, Tempe, Arizona, USA; d Center for Evolution and Medicine, Arizona State University, Tempe, Arizona, USA; e Structural Biology Research Unit, Department of Clinical Laboratory Sciences, University of Cape Town Observatory, Cape Town, South Africa; DOE Joint Genome Institute

## Abstract

Complete genome sequences of two novel torque teno viruses (TTVs) were identified in human brain tissue. These sequences are 3,245 nucleotides (nt) and 2,900 nt long and share 68% and 72% open reading frame 1 (ORF1) identity, respectively, with other human TTVs. This report extends the identification of TTV sequences in the brain.

## ANNOUNCEMENT

Since the discovery of the first torque teno virus (TTV) (family *Anelloviridae*) from a human sample, a plethora of anelloviruses have been identified in various mammals (including primates) and avian species. *Anelloviridae* is a family of host-specific diverse viruses (1–5). In primates, a number of TTV genome sequences have been identified from a broad range of tissue and sample types (5–8). Studies in humans have investigated associations between TTV and outcomes of diseases such as cancer and hepatitis (8–10). Previously, two TTV genome sequences were identified in a glioblastoma sample (GenBank accession no. KX810063 and KX810064) (11), and Tisza et al. (12) reported the identification of anelloviruses (partial genomes) in the brain tissue of deceased individuals with Alzheimer’s disease and other forms of dementia.

Given that only two genome sequences of TTV have been identified from brain tissue samples, we undertook a viral metagenomic analysis of human brain tissue to identify additional TTVs. Written informed consent for autopsy was obtained in compliance with institutional guidelines of Banner Sun Health Research Institute. The Banner Sun Health Research Institute Review Board approved this study, including recruitment, enrollment, and autopsy procedures. Individual person(s) and their respective next of kin consented to brain autopsy for the purpose of research analysis as participants in the Banner Sun Health Research Institute Brain and Body Donation Program. The human brain tissue used in this study was from routine existing autopsies, which fully qualifies for 4C exemption by NIH guidelines.

**FIG 1:**
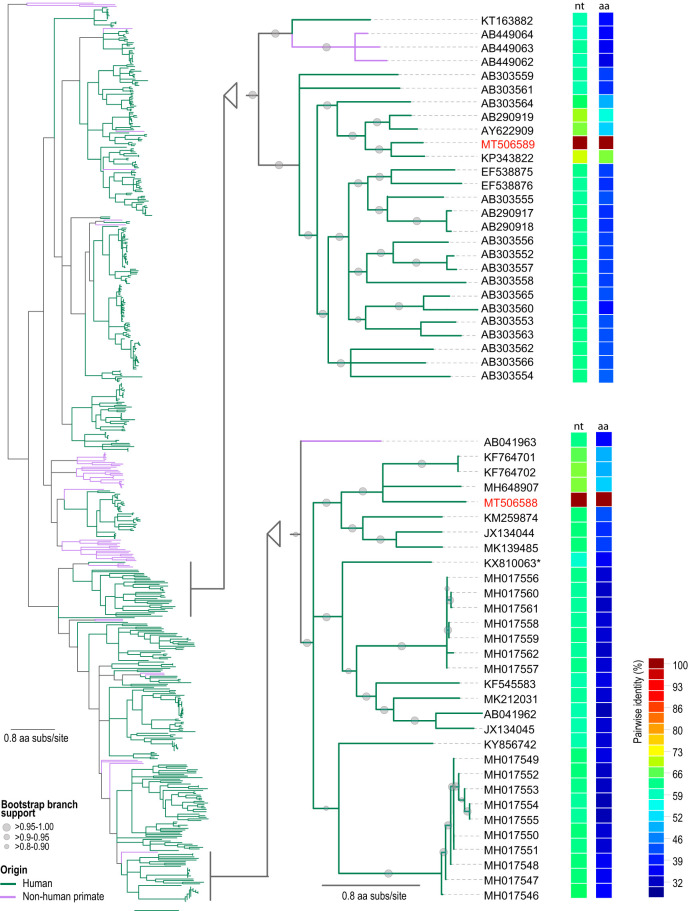
Maximum likelihood phylogenetic tree of primate anellovirus ORF1 protein sequences. The clades that have the two anelloviruses (GenBank accession no. MT506588 and MT506589; red font) reported here from the human brain sample are shown in detail. Branches with <0.8 approximate likelihood ratio test (aLRT) support have been collapsed using TreeGraph2 (21). Pairwise identity of the ORF1 gene (nt) and protein sequences (aa) in relation to MT506588 and MT506589 in the two separate clades are shown to the right of the accession numbers. *, anellovirus sequence from glioblastoma sample (11).

Brain tissue of the middle temporal gyrus (0.1 g) from an 82-year-old deceased individual (identifier [ID] 04-05) with no abnormal brain pathology was homogenized in 300 μl of SM buffer (0.1 M NaCl, 50 mM Tris-HCl [pH 7.4], 10 mM MgSO_4_), and viral DNA was extracted from the supernatant using the High Pure viral nucleic acid kit (Roche Diagnostics, USA). Circular DNA was preferentially amplified by rolling-circle replication amplification (RCA) with the TempliPhi kit (GE Healthcare, USA) and random hexamers. The resulting RCA DNA was used to generate 2 × 100-bp libraries using the Nextera DNA flex kit (Illumina, USA) and was sequenced at Macrogen, Inc. (South Korea), on the Illumina HiSeq 4000 platform. Raw reads were *de novo* assembled using metaSPAdes v3.12.0 (13), and the resulting contigs were analyzed using BLASTx against a viral database for similarities to viral proteins (14). Two contigs (1,964 and 3,079 nucleotides [nt]) with similarity to anelloviruses were identified. We designed specific abutting primers (Table 1) which were used to recover the complete genome sequences by PCR with the HiFi HotStart DNA polymerase (Kapa Biosystems, USA). Genomes were cloned and Sanger sequenced at Macrogen, Inc. The two recovered viral genome sequences (GenBank accession no. MT506588 and MT506589) are 2,900 nt (37.2% GC content) and 3,245 nt (42.5% GC content) long and have an organization typically exhibited by anelloviruses. Since the largest open reading frame 1 (ORF1) is relatively conserved, we assembled a data set of primate anellovirus ORF1 sequences available in GenBank (downloaded on 1 April 2020). A maximum likelihood phylogeny of the MUSCLE v3.8.31 (15)-aligned sequences of the ORF1 proteins was constructed using PHYML v3.0 (16) (substitution model rtREV+G+F) (17). Nucleotide and amino acid pairwise identities were determined using SDT v1.2 (18). The analysis (Fig. 1) shows that the two TTVs identified from the brain sample fall into two distinct clades and share the highest similarity with other human TTVs. The sequence under accession no. MT506588 is most closely related to three anelloviruses derived from human blood, under accession no. KF764701, KF764702 (19), and MH648907 (12), shares ∼68% ORF1 gene pairwise identity, and is a member of the *Betatorquevirus* genus (Fig. 1). The sequence under accession no. MT506589 is most closely related to a sequence from sera taken from a first-trimester mother (KP343822) (20), shares ∼72% ORF1 gene pairwise identity, and is member of the *Gammatorquevirus* genus (Fig. 1). The sequences under accession no. MT506588 and MT506589 extend our knowledge of TTV diversity in humans and complement the two genome sequences previously identified in the brain samples (11).

**TABLE 1:** Details of primers used to amplify the TTV genome

Sequence accession no.	Primer pair
MT506588	5′-AGATGTAGACTTAATACAAGCAGGCCTACCAC-3′; 5′-GTTGGGAAGCTCCATTCGTCTGTGTAATC-3′
MT506589	5′-TCACCTGCTTGCTACTATTTTTCCTCCTGG-3′; 5′-GCAAAAGGATGGTCGCAGTCACAGATATTG-3′

### Data availability.

The TTV sequences have been deposited in GenBank under the accession no. MT506588 and MT506589. Mapped raw reads have been deposited in the SRA database under SRR12450126.
